# A rare case of endometrioma in a bitch

**DOI:** 10.1186/s13028-015-0123-1

**Published:** 2015-06-18

**Authors:** Bruno H. A. Paiva, Juneo F. Silva, Natália M. Ocarino, Cleida A. Oliveira, Wiviane A. Assis, Rogéria Serakides

**Affiliations:** Laboratório de Patologia do Departamento de Clínica e Cirurgia Veterinárias, Escola de Veterinária da Universidade Federal de Minas Gerais, Avenida Presidente Antônio Carlos, 6627, CEP: 30.161-970, Belo Horizonte, Minas Gerais Brazil; Laboratório de Biologia da Reprodução do Departamento de Morfologia, Instituto de Ciências Biológicas da Universidade Federal de Minas Gerais, Avenida Presidente Antônio Carlos, 6627, CEP: 30.161-970, Belo Horizonte, Minas Gerais Brazil

**Keywords:** Endometriosis, Endometrioma, Coelomic metaplasia, Bitch

## Abstract

**Background:**

Endometriosis is ectopic development of endometrial glands and stroma in extra-uterine sites and if the lesions occur as a well-defined mass is referred to as endometrioma. In the literature, endometrioma has been reported in only women and there are no reports of endometrioma in animals, including non-human primates.

**Case presentation:**

A rare case of endometrioma is reported in an 11-year-old female German Shepherd with clinical signs of dehydration, anemia and prostration. Necropsy revealed ascites, generalized pallor, and a well-demarcated reddish mass adjacent to the left ovary and uterus and adherent to the retroperitoneum. The mass measured 25.0 × 20.0 cm with intermingled soft and firm areas. Upon incision, the mass was found to be solid with variable sized cystic cavities filled with coagulated blood. Microscopically, the mass was composed of cuboidal or prismatic epithelial cells arranged in tubules or acini. The epithelium of the mass had similar characteristics to the normal endometrium with PAS-positive secretions. The stroma was prominent and formed by loose connective tissue and smooth muscle fibers as confirmed by Masson trichrome. Extensive multifocal areas of hemorrhage were also observed in the stroma of the mass and in the interior of some epithelium-lined, cystic structures. Most of the epithelial cells had strong and diffuse cytokeratin expression, and some had vimentin expression. Epithelial and stromal cells also showed ERβ, AR, VEGF and COX2 expression. The stroma showed areas with strong and diffuse vimentin expression. Factor VIII expression was observed only in the endothelium of blood vessels in the stroma.

**Conclusions:**

The macroscopic, microscopic and immunohistochemical findings are consistent with an endometrioma.

## Background

Endometriosis is ectopic development of endometrial glands and stroma in extra-uterine sites [1, 2]. Endometriosis is referred to as endometrioma if the lesions occur as a well-defined mass between 2 and 15 cm in diameter. An endometrioma consists of endometrial glands, stroma and blood, and is mostly located adjacent to the ovary or the abdominal wall [3].

Endometriosis is a common gynecological disease in women, affecting approximately 10 % of the female population in the reproductive age. In rare cases, endometriosis has been diagnosed in men [4, 5]. In animals, the natural occurrence of endometriosis has been reported in only primates such as gorillas, cynomolgus monkeys, rhesus monkeys and baboons [6]. In the literature, endometrioma has been reported in only women [7, 8]. There are no reports of endometrioma in animals, including non-human primates.

Two theories have been proposed to explain the development of endometriosis in women and non-human primates. The retrograde menstruation theory suggests that viable endometrial cells, which are shed during menstruation, are fixed outside of the uterus [1]. The coelomic metaplasia theory is based on the pluripotent characteristic of the parietal peritoneum, as it is the surface epithelium of the ovary and the remnants of the Müllerian ducts [9–12]. The transplantation theory hypothesizes that endometriosis occurs by iatrogenic implantation of endometrial tissue in the skin after cesarean surgery [13]. Estradiol plays an important role in the appearance, perpetuation and proliferative potential of endometrial tissue; therefore, it is therefore widely used to induce endometriosis in animal models [14–16].

The occurrence of endometriosis in animal species with an estrous cycle is rare. In addition to the lack of shedding of the endometrium, which prevents the development of endometriosis by implantation, these species have minimum levels of estradiol, with only one peak along the estrous cycle [17]. Therefore, the occurrence of endometriosis in the dog can be attributed to the coelomic theory, which is most likely to explain the development of endometriosis in women outside of the reproductive age and in men [18–20].

The objective of this study is to report the macroscopic, microscopic and immunohistochemical features of an endometrioma in a bitch.

## Case presentation

An 11-years-old female German Shepherd was admitted to the Veterinary Teaching Hospital of the Universidade Federal de Minas Gerais, Brazil with a history of prostration one month ago. According to the owner, the animal had multiple normal deliveries, never exposed to contraceptives and experienced estrus regularly twice a year. Clinical examination showed mild dehydration, prostration and intensely pale mucosa. However, no other changes were observed as the dog suddenly died during the clinical examination, before laboratory testing was possible.

**Fig. 1:**
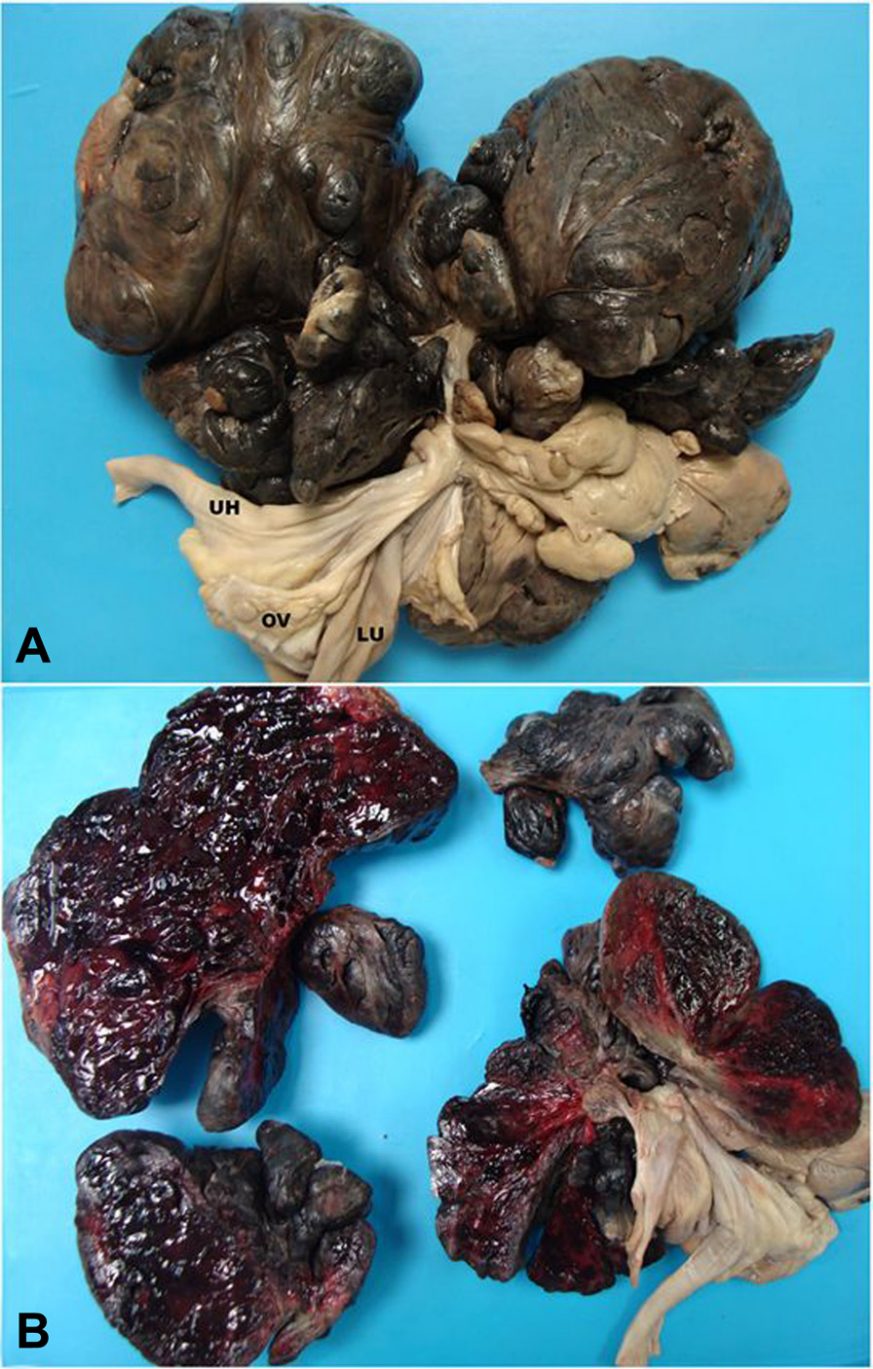
Gross lesions in case of endometrioma in a bitch. **a** Adjacent to the left ovary and uterus, the presence of a well-defined reddish mass, measuring 25.0 × 20.0 cm. **b** Upon incision, the mass was found to be solid with cavitary formations of varying sizes filled with coagulated blood (UH - Horn uterine; LU - broad ligament of the uterus; OV - Ovary)

Necropsy revealed intensely pale mucous membranes, generalized atrophy of the skeletal muscles and absence of subcutaneous and visceral adipose tissue. In the abdominal cavity, there were approximately 600 ml of reddish and translucent fluid. Adjacent to the left ovary and uterus and adherent to the retroperitoneum, there was a well-demarcated reddish mass, measuring 25.0 × 20.0 cm (Fig. 1a) with a mixture of soft and firm areas. Upon incision, the mass was found to be solid with variable sized cystic cavities filled with coagulated blood (Fig. 1b). The lungs showed moderate congestion. The other organs showed no significant lesions.

The entire genital tract and specimens of the mass and other abdominal and thoracic organs were fixed in 10 % neutral phosphate-buffered formalin, sectioned, processed, embedded in paraffin, and stained with hematoxylin-eosin for histopathological examination. To better characterize the mass, staining with Masson trichrome, Periodic Acid Schiff (PAS) and immunohistochemistry were performed. Antibodies used for immunohistochemistry and their dilutions were as follows: anti-Factor VIII (1:300) (A0082, DAKO, St. Louis, MO, USA), anti-vimentin (1:200) (Sigma Chemical Co. St. Louis, MO USA), anti-cytokeratin (1:200) (AE1, AE3, Dako, St. Louis, MO, USA), anti-VEGF (1:100) (sc-152, Santa Cruz Biotechnology, CA, USA), anti-COX2 (1:40) (M3617, Dako, St Louis, MO, US), anti-estrogen receptor (ER) β (1:25) (Novocastra Laboratories, Newcastle, UK), and anti-androgen receptor (AR) (1:500) (PG21, Upstate, Lake Place, NY). The streptavidin-biotin-peroxidase technique was used and antigen retrieval was performed in a water bath at 98 °C using Retrieval solution for anti-Factor VIII, anti-cytokeratin and anti-vimentin antibodies. For anti-ERβ and anti-androgen receptor (AR), antigen retrieval was achieved by microwave. The slides were incubated overnight in a humid chamber with the primary antibody and for 30 min for each of the following steps: blocking endogenous peroxidase, blocking serum (Ultra Vision Block; Fremont, CA, USA) and streptavidin peroxidase (Vectastain Elite ABC Kit, Vector Laboratories, Burlingame, USA for anti-ERβ and anti-androgen receptor and LSAB kit, Dako, St. Louis, MO, USA for others antibodies). Incubation with the secondary antibody (Dako, St. Louis, MO, USA) was performed for 45 min. The chromogen used was diaminobenzidine (DAB Substrate System, Dako, St. Louis, MO, USA). Sections were counterstained with Harris hematoxylin. As a positive control for each antibody, the ovary of the animal was used because it expresses all the markers assessed. A negative control was performed by replacing the primary antibodies by IgG.

**Fig. 2:**
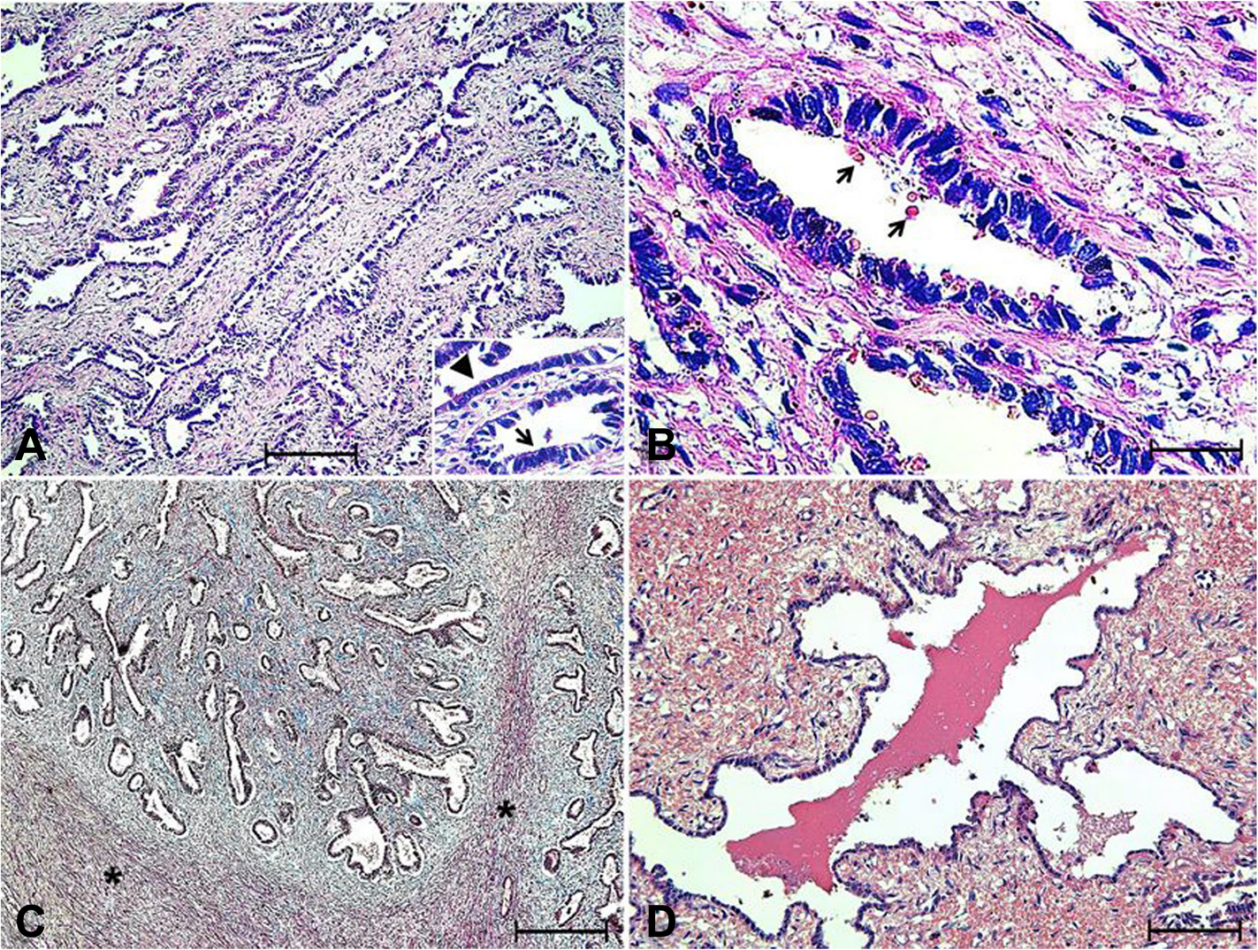
Microscopic lesions in case of endometrioma in a bitch. **a** Endometrioma presenting epithelial tissue in detail (arrow) similar to the endometrium forming tubules, acini or cavity structures filled with blood and surrounded by intense stroma (Hematoxylin and eosin, bar = 250 μm). **b** Epithelium with particulate and eosinophilic material at the apical surface, consistent with secretion (Periodic Acid-Schiff, bar = 24 μm). **c** Fibers of smooth muscle forming the stroma of the endometrioma (asterisk) (Masson trichrome, bar = 250 μm). **d** Extensive areas of bleeding in the stroma and within the lumen of tubules coated with endometrial epithelium (Hematoxylin and eosin, bar = 250 μm)

Microscopically, the mass was composed of cuboidal or prismatic epithelial cells arranged in tubules, acini or cystic structures, which were sometimes filled with erythrocytes. The epithelium had a round basal nucleus with loose chromatin and the apex of the cell had a finely particulate PAS-positive material (Fig. 2a and b). The epithelium of the mass had similar characteristics to that of a normal endometrium. No mitotic figures or cellular atypia were observed. The stroma surrounding the epithelial tissue was prominent and formed by loose connective tissue and smooth muscle fibers stained red with Masson trichrome staining (Fig. 2c). Extensive multifocal areas of hemorrhage were also observed in the stroma of the mass and blood was present in the lumen of some cystic structures lined with endometrial epithelium (Fig. 2d).

**Fig. 3:**
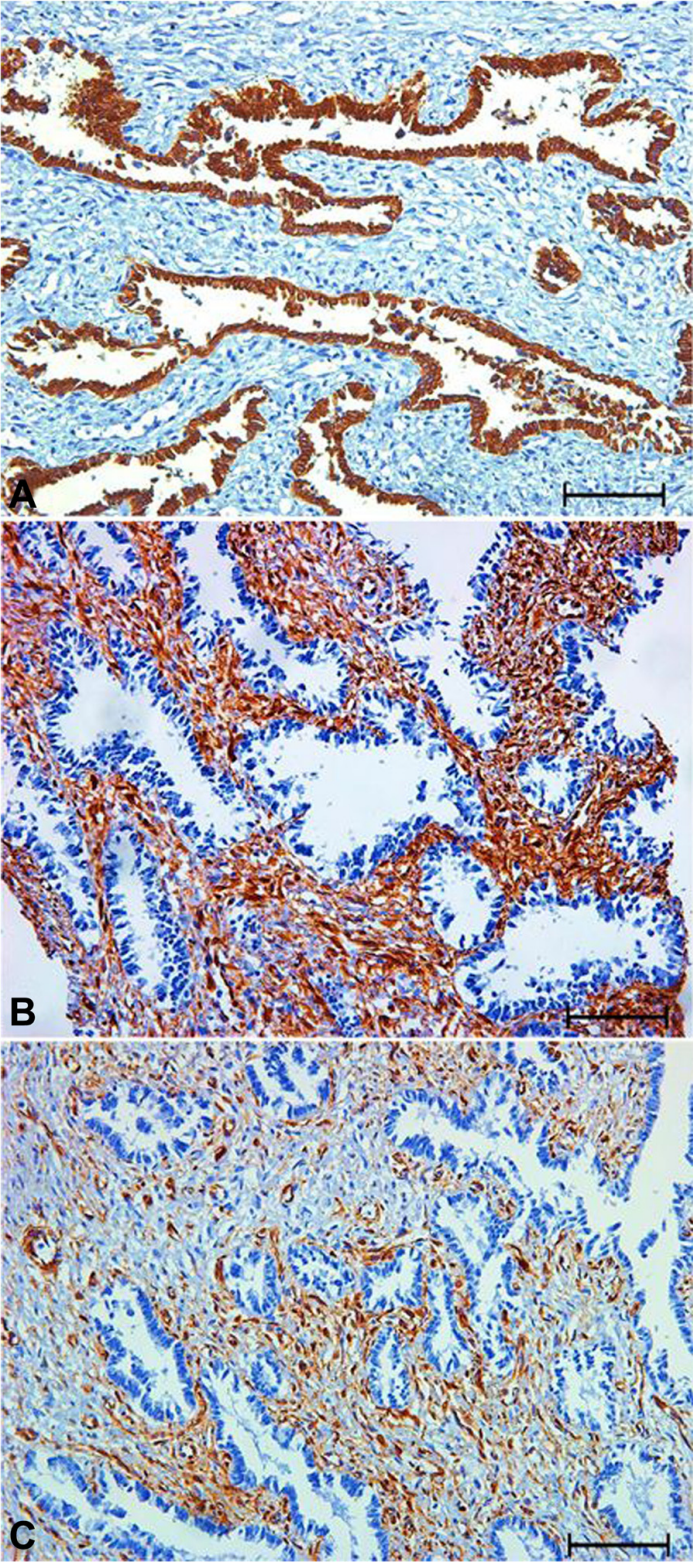
Cytokeratin and vimentin immunohistochemistry, endometrioma, bitch. **a** Cytokeratin immunostaining showing intensely labeled areas in the epithelium. **b** and **c** Vimentin immunostaining showing intensely labeled areas in the stroma (**b**) and other areas with lower expression (**c**). (Streptavidin-biotin-peroxidase method, Harris’ hematoxylin counterstain, bar = 225 μm)

**Fig. 4:**
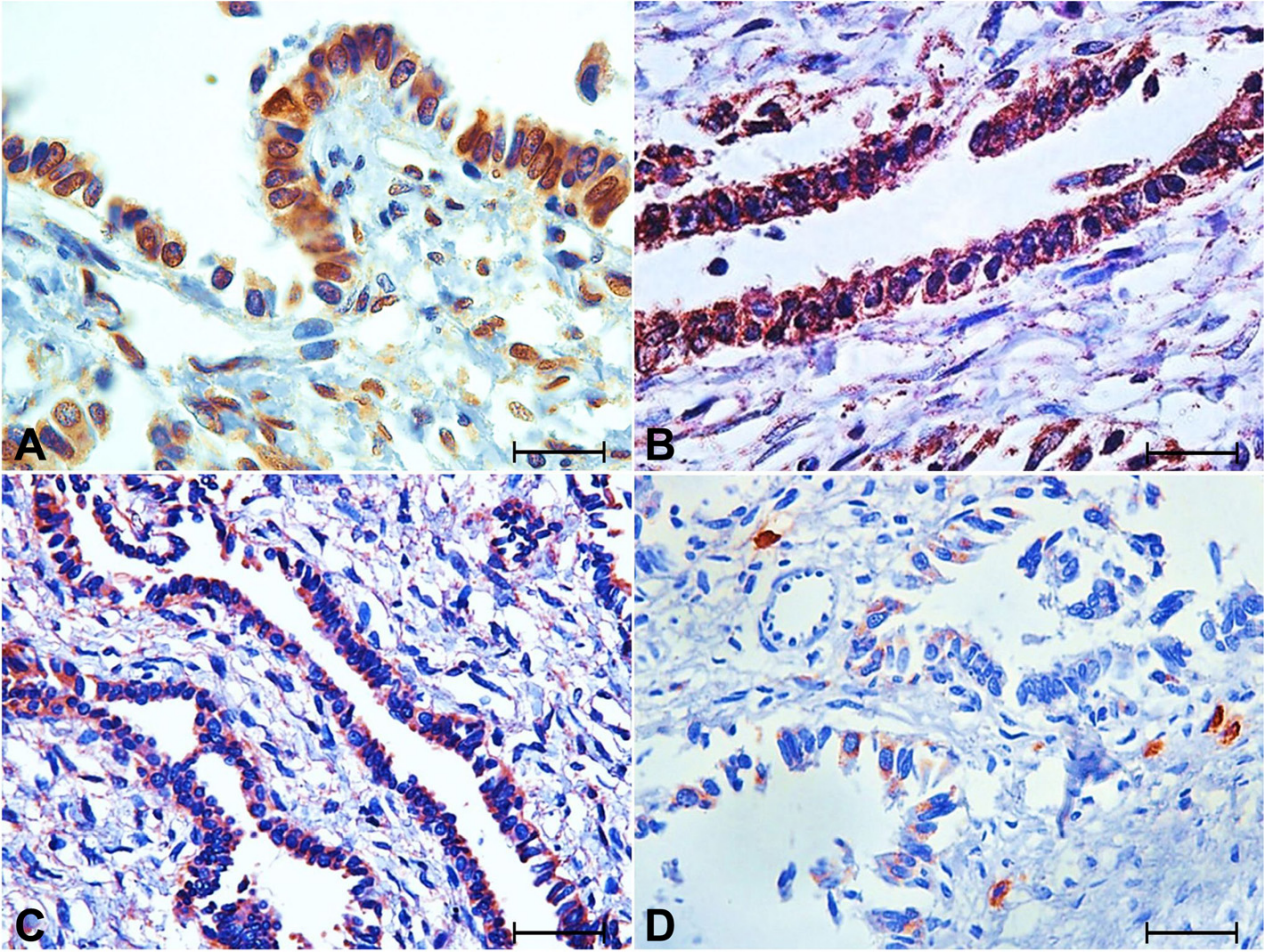
Immunohistochemical staining for ERβ, androgen receptor, VEGF and COX2, endometrioma, bitch. **a** and **b** ERβ (**a**) and androgen receptor (AR) (**b**) immunostaining showing the majority of the epithelial cells and some stromal cells with nuclear and cytoplasmic expression. **c** VEGF immunostaining showing epithelial cells, endothelium of blood vessels and some stromal cells with cytoplasmic and strong expression. **d** COX2 immunostaining showing some epithelial cells with moderate expression and some stromal cells with strong expression (Streptavidin-biotin-peroxidase method, Harris’ hematoxylin counterstain, bar = 48 μm (**a**, **b**); 96 μm (**b**, **c**))

Epithelial cells, especially prismatic cells, formed structures such as endometrial glands and demonstrated strong and diffuse cytokeratin expression (Fig. 3a) although some cuboidal and flattened epithelial cells demonstrated weaker cytokeratin expression. The stroma possessed areas with strong vimentin expression between areas with weaker expression (Fig. 3b and c). Some epithelial cells also displayed vimentin expression. The majority of the epithelial cells and some stromal cells also possessed cytoplasmic and nuclear ERβ and AR expression (Fig. 4a and b). VEGF expression was cytoplasmic, strong and diffuse in epithelial cells, the endothelium of blood vessels, and in some stromal cells (Fig. 4c), whereas COX2 expression was moderate in some epithelial cells and strong in some stromal cells (Fig. 4d). Factor VIII expression was observed in only the blood vessels of the stroma.

Moderate cystic endometrial hyperplasia with the presence of endometrial glands in the myometrium was observed in the uterus (adenomyosis). The right and left ovaries showed corpus luteum in regression and some follicles at different stages of development. The other organs showed no significant microscopic changes.

Clinical and macroscopic findings of anemia may have been derived from extensive bleeding in the endometrial tissue. Cases of ascites associated with endometriosis have also been reported. It is suggested that the occurrence of ascites may be due to the rupture of cysts in the endometrial tissue with extravasation of fluid and red blood cells into the abdominal cavity. Moreover, when the formation of large masses occurs, as in this dog, lymphatic drainage may be impaired with extravasation of fluid from the endometrial tissue or from adjacent organs and tissue [8, 21–23]. Although rare, endometriosis in women may cause ascites, anemia and circulatory shock [24].

Despite the endometrioma, the dog had showed no disturbance of the estrous cycle or fertility as it had delivered normal viable full-term offspring previously. Unlike endometrioma, deep endometriosis is the main presentation that causes pelvic pain and infertility in women [25, 26]. In women, there are also reports of large endometriomas during pregnancy [25, 27].

The occurrence of endometriosis caused by cell implantation is unlikely in dogs because dogs lack the endometrial desquamation observed in women during menses and in non-human primates in the diestrus phase. In dogs, endometrial shedding does not occur and the bleeding during estrus comes from red cell diapedesis from blood vessels. In addition, female dogs have 3–10 months of anestrus characterized by the permanence of serum sex hormones at basal levels. Therefore, we suggest that these differences might favor, at least in part, the occurrence of endometriosis in women [28]. However, the coelomic mesothelium is a pluripotent tissue with high transformation capacity. In bitches, morphological changes of the coelomic mesothelium as cysts and tumors are relatively common compared to other species. Therefore, the coelomic metaplasia theory of the parietal peritoneum better supports the development of the ectopic endometrial tissue in this case [9, 11, 12]. This theory has also been suggested to explain the genesis of endometriosis in men and women outside of reproductive age [18, 19, 29] as well as endometriosis in distant extra-pelvic organs [30].

Studies have demonstrated that estradiol plays an important role in the appearance, perpetuation and proliferative potential of the endometrial tissue [10, 14, 16]. In humans, some cases of endometriosis are associated with prolonged use of estradiol for the treatment of prostate cancer [31]. However, the bitch in this case did not receive hormonal treatment or had been exposed to contraceptives. In addition, no hormonally active ovarian alteration was observed. However, we cannot rule out the involvement of estradiol in the pathogenesis of the endometrioma in this bitch, mostly because the ectopic endometrial tissue showed immunohistochemical expression of the β receptor for estradiol.

Despite the macroscopic characteristics of the mass described in some cases of endometrioma in women [7, 32], based solely on the macroscopic mass, especially the red color and the presence of cavities filled with coagulated blood, an initial diagnosis of hemangiosarcoma was made in this dog. However, microscopic and immunohistochemical features belied this suspicion. The histomorphology was slightly similar to cystadenoma of the *rete ovarii* because such tumors have cysts averaged 8.7 cm, a flat, cuboidal, or columnar, usually nonciliated-lining epithelium and can sometimes present a fibromuscular wall [33, 34]. However, the cysts are not filled with blood and there is not extensive bleeding in the stroma as seen in this case [33, 34]. The extra-ovarian location and the microscopic characteristics of the mass also ruled out the possibility of a granulosa cell tumor or ovarian germ cell tumor [35, 36]. The differential diagnosis of cystic abdominal mesothelioma or others celomic epithelium tumors such as ovary papillary cystadenoma and cystadenocarcinoma were also ruled out. Abdominal mesothelioma in dogs generally presents by dissemination to the parietal and visceral peritoneum [37]. However, the human peritoneal mesothelioma may also be present as a major pelvic mass [38]. Moreover, mesothelioma cysts are characterized by the presence of bright and clear yellow fluid, contrary to what was observed in the present dog. The epithelial tissue of the mass was also positive for PAS secretion, and the stroma had smooth muscle cells, which are not found in mesothelioma and ovary papillary cystadenoma [39–42].

The endometrial tissue found in this bitch was restricted to a mass located adjacent to the uterus and ovary and fixed to the retroperitoneum. The association of the epithelium with a richly vascularized fibrous stroma observed in the present dog has also been described in endometrioma in women [3, 7, 43]. However, the presence of bundles of smooth muscle in the stroma of the lesion, as was observed in this case, is unusual. This feature makes the lesion more of a uterus-like mass or endomiometrioma [44].

The immunohistochemical characteristics of endometriosis have been widely studied. Similar to what was observed herein, ectopic endometrial cells are positive for cytokeratin [45] and may also express receptors for estradiol and androgen [46, 47]. In this case, some endometrial cells also expressed vimentin. The endometrium is derived from intermediate mesoderm through the transition from the epithelium to mesenchyme during development of the urogenital system. Although vimentin is a mesenchymal marker, it is expressed in epithelial tissues derived from mesoderm, such as endometrium [45]. Cytokeratin expression in epithelial cells was diffuse, as opposed to vimentin expression, which was multifocal and less intense. This difference can be attributed to the degree of cell differentiation of the epithelium, as cytokeratin and vimentin expression varies with the composition of the cell filaments and, consequently, the degree of histological differentiation [11, 48].

Immunohistochemical expression of COX2 and VEGF by endometrial epithelium and stromal cells was also observed in cases of endometriosis in women [49, 50]. It is believed that the expression of COX2 and VEGF, stimulated by estradiol, promote the growth and maintenance of endometrial tissue to stimulate angiogenesis and, consequently, the blood supply [7, 51–53]. Inhibition of COX2 in rats with experimental endometriosis causes regression of endometrial tissue by inhibiting VEGF expression and angiogenesis [54, 55]. The endometrioma observed in this dog was highly vascularized, which was important to maintain tissue viability as areas of necrosis were not observed despite the large size of the mass.

## Conclusions

Based on the macroscopic, microscopic and immunohistochemical findings, the diagnosis of endometrioma was confirmed. The occurrence of endometrioma in animal species with an estrous cycle is rare, and this is the first case described in dogs.
